# Supplementary material for: Utility of red‐light ultrafast optogenetic stimulation of the auditory pathway
Source: EMBO Mol Med. 2021 May 7;13(6):e13391. doi: 10.15252/emmm.202013391 (PMC8185542; doi:10.15252/emmm.202013391)
Supplement: Supplementary file 1 — Expanded View Figures PDF [file EMMM-13-e13391-s004.pdf]

Expanded View Figures

Figure EV1. Expression analysis of vf-Chrimson-eYFP and f-Chrimson-eYFP expressed in NG108-15 cells.

A–F Immunofluorescence of f-Chrimson-eYFP (A, C, E) and vf-Chrimson-eYFP (B, D, F) plotted across the plasma membrane and the membrane proximal intracellular regions. The line profiles are obtained from single-stack confocal images of NG cells transfected with either construct ($n = 30$ cells, $N = 4$ transfections per construct). Line profiles are grouped according to the cellular distribution of the fluorescence. (A + B): intracellular fluorescence weaker than plasma membrane fluorescence, (C + D): intracellular fluorescence similar to plasma membrane fluorescence (E + F): gradual increase of fluorescence from plasma membrane to cell interior.

G Exemplary live cells, single-stack confocal image of NG cells expressing f-Chrimson-eYFP. Scale bar = 10 μm.

H Bar graph summarizing the line profiles, grouped according to the cellular distribution of the fluorescence shown in (A-F).

 © 2021 The Authors

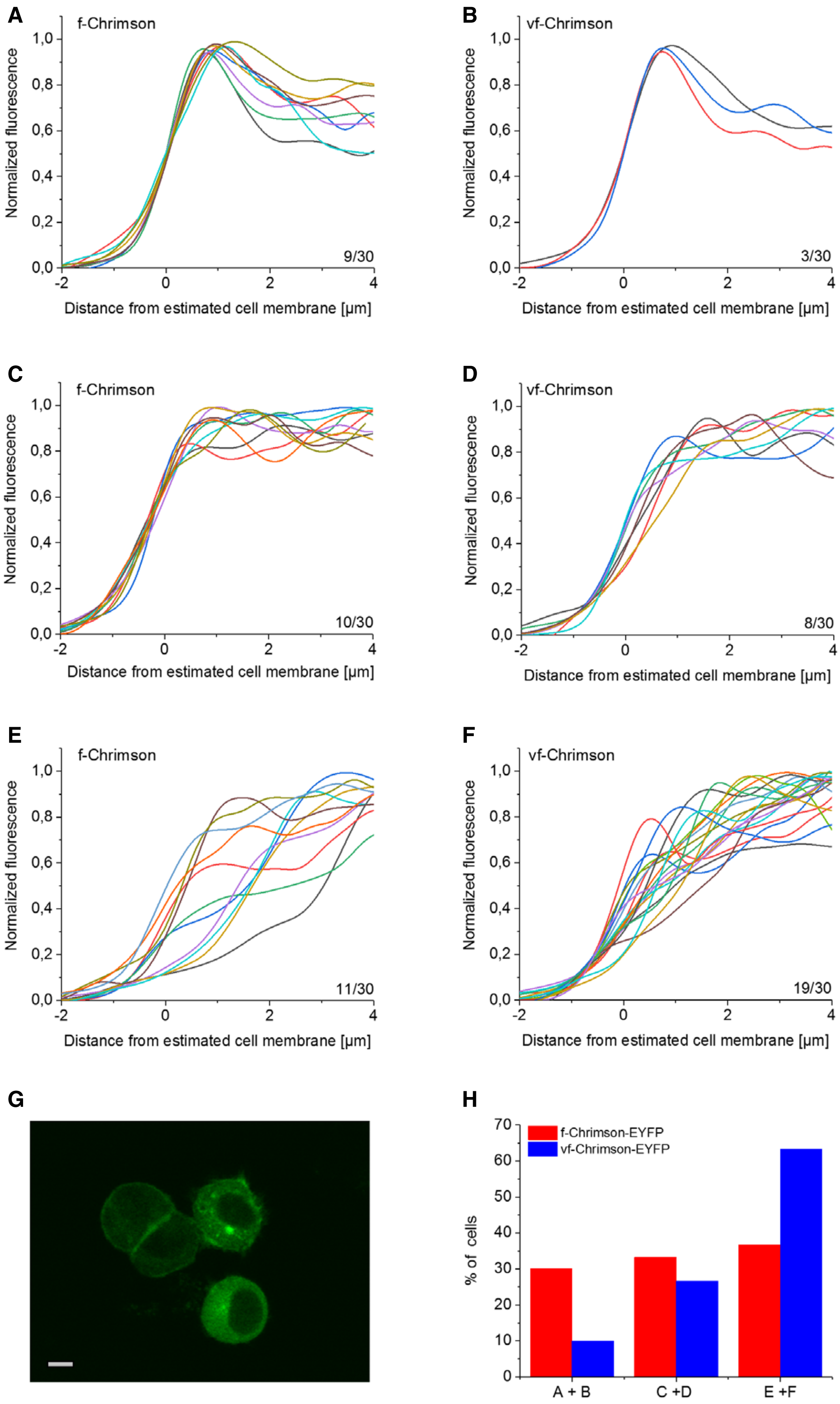
A
f-Chrimson
Normalized fluorescence
9/30
Distance from estimated cell membrane [µm]
B
vf-Chrimson
3/30
C
f-Chrimson
10/30
D
vf-Chrimson
8/30
E
f-Chrimson
11/30
F
vf-Chrimson
19/30
G
H
f-Chrimson-EYFP
vf-Chrimson-EYFP
% of cells
A + B
C +D
E +F

Figure EV1.

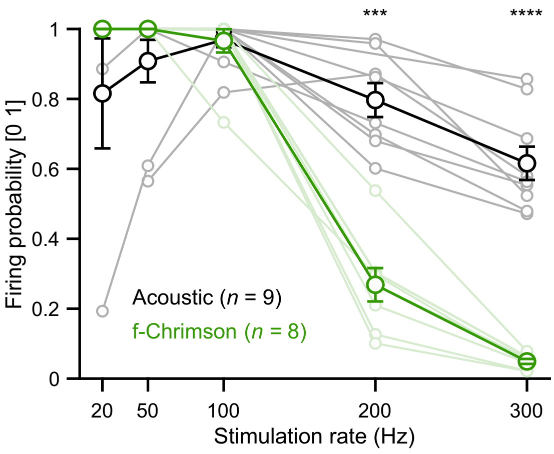

Figure EV2. Firing probability (i.e., probability of observing an action potential evoked by the click/light pulse) decreases with increasing stimulation rates of acoustic and optogenetic stimulation (f-Chrimson).

Firing probability as a function of the stimulation rate (mean ± SEM) measured acoustically from naïve putative SGNs (black, 100 dB SPL (pe), 300 μs acoustic click, $n = 9$ units, $N = 3$ mice) or optically from f—Chrimson expressing SGNs (green, 18.3 mW, 1-ms pulse length; $n = 8$, $N = 2$ mice). Stimulation was composed of a 400 ms click/light pulse trains followed by 100-ms recovery in silence/dark. Wilcoxon rank-sum test (***: P-value $\leq 10^{-3}$, ****: P-value $\leq 10^{-4}$).

© 2021 The Authors
